# Development of a rapid recombinase polymerase amplification-lateral flow dipstick assay for sensitive detection of duck adenovirus type-3

**DOI:** 10.3389/fmicb.2025.1638182

**Published:** 2025-08-13

**Authors:** Jiayu Sun, Kewei Liu, Chao Liu, Zhenyu Wang, Jiaxi Gao, Ruya Zhao, Long Yuan, Yan Shen, Jinchun Li, Fangfang Chen

**Affiliations:** ^1^Zoonoses Laboratory and Key Laboratory of Veterinary Pathobiology and Disease Control, College of Veterinary Medicine, Anhui Agricultural University, Hefei, China; ^2^Anhui Animal Disease Prevention and Control Center, Hefei, China

**Keywords:** DAdV-3, RPA-LFD, detection, clinical diagnosis, specificity

## Abstract

Duck adenovirus type-3 (DAdV-3) infections have severe effects on duck health, and rapid detection methods are crucial to reduce the morbidity and mortality associated with this pathogen in clinical practice. In this study, we established, optimized, and validated a novel recombinase polymerase amplification (RPA)-lateral flow dipstick (LFD) assay for the detection of DAdV-3. Next, we established a clinical infection model based on the pathogenicity of the DAdV-3 strain and tested the effectiveness of the RPA-LFD assay in controlling an outbreak of DAdV-3 infection. The findings indicated that the RPA-LFD assay could be performed within 30 min at 42°C. Specificity tests indicated no cross-reactivity with other viruses. The detection limit of the assay was 1 × 10^1^ copies/μL. We evaluated 65 clinical samples using RPA-LFD and quantitative polymerase chain reaction (qPCR), and both methods showed a positivity rate of 33.8% and a coincidence rate of 100%. The kappa (*κ*) value of the RPA-LFD and qPCR assays was 1 (*p* < 0.001). The application of this assay in experimentally infected ducklings reduced the mortality rate from 20 to 8%. Thus, the RPA-LFD assay established in this study demonstrated high specificity, sensitivity, rapidity, and efficacy, indicating its potential for rapid detection of DAdV-3 in clinical settings.

## 1 Introduction

Aviadenovirus is a non-enveloped DNA virus with seven currently known species that can infect chickens or waterfowl, namely, fowl adenovirus (FAdV) A, B, C, D, and E and Duck adenovirus (DAdV) A and B (Hess et al., 1997; Niu et al., 2018; Schachner et al., 2018; Zhuang et al., 2023). Some serotypes, such as FAdV-1, 4, 8a, 8b, 11, DAdV-1 (egg-drop syndrome virus, EDSV), and DAdV-3, can exhibit cross-infection between chickens and waterfowl but produce different clinical signs and pathological changes (Kang et al., 2017; Ono et al., 2001; Wu et al., 2024; Zhang X. et al., 2024; Zhuang et al., 2023). However, DAdV-3 primarily affects Muscovy ducks, which exhibit liver bleeding, pericardial effusion, and renal swelling (Zhang et al., 2016). Recent studies have shown that the virus can also infect chickens, mainly causing hepatitis-hydropericardium syndrome (HHS), bursa necrosis, and disintegration of the gizzard endothelium (Zhang X. et al., 2024). However, some strains (HF-AN-2020) did not cause obvious clinical symptoms in ducklings but induced interferon (IFN) production in animals (Tan et al., 2024). Significant differences have been observed in the virulence of different strains of DAdV-3, with the primary amino acid differences identified in ORF19B, ORF66, and ORF67 (Tan et al., 2024; Zhang X. et al., 2024). In veterinary clinical practice, timely detection can reduce the morbidity and mortality rates associated with DAdV-3 infection.

The DAdV-3 serotype belongs to DAdV-B and is the most severe adenovirus that affects waterfowl. The virions of DAdV-3 are non-enveloped and spherical, with an icosahedral symmetry, and a diameter of 80 nm. The total genome length of the virus is 43,841 bp (Shi et al., 2022). All Aviadenovirus capsids include penton and hexon; the former contains a penton base and fibers, and DAdV-3 has two fiber proteins, fiber-1 and fiber-2, with amino acid homology of 58.42% (Benko et al., 2022; Marek et al., 2014). The distinct hexon amino acids of different Aviadenovirues form the basis of virus typing; in contrast, the fiber protein is relatively conserved and is the main domain for virus invasion of the host; therefore, it is a key target for virus detection and the development of subunit vaccines (Lin et al., 2023; Liu et al., 2024; Marek et al., 2010; Shao et al., 2022; Yin et al., 2019). Although both *fiber-1* and *fiber-2* are conserved among DAdV-3 strains with different virulence, *fiber-2* has a longer coding sequence than *fiber-1*. Moreover, it exhibits lower homology with fiber proteins from other aviadenoviruses, making it more suitable as a target for DAdV-3 detection. The existing methods for the detection of DAdV-3 include polymerase chain reaction (PCR), quantitative PCR (qPCR), indirect enzyme-linked immunosorbent assay (ELISA), and loop-mediated isothermal amplification (LAMP) (Chen et al., 2019; Kaján et al., 2011; Wan et al., 2018; Xie et al., 2011). The first three methods require special instruments and professional operators, which limits their application in aquaculture farms (Asiello and Baeumner, 2011). However, approaches based on these methods cannot simultaneously meet the requirements of high specificity and simple clinical operation. Although the LAMP method enables rapid amplification at 60–65°C, it has high requirements for primers and is not easy to operate (Li et al., 2024). Thus, there is an urgent need for a rapid, specific, and on-site detection method to address these limitations.

**Figure 1:**
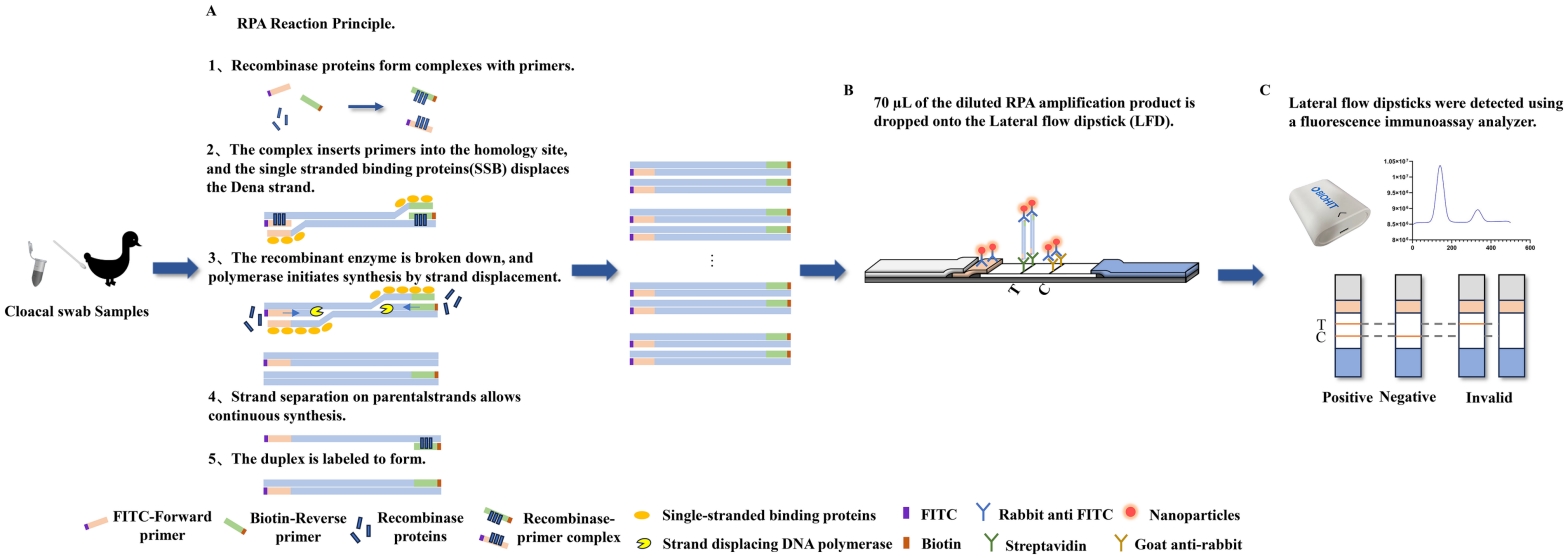
The basic principle underlying the detection of DAdV-3 through the RPA-LFD assay. **(A)** The recombinase polymerase amplification (RPA) reaction was applied to amplify double-stranded DNA fragments labeled with fluorescein isothiocyanate (FITC) and biotin. **(B)** 70 μL of the diluted RPA amplification product is dropped onto the lateral flow dipstick (LFD). Rabbit anti-FITC antibody was conjugated to fluorescent microspheres and subsequently applied onto the conjugate pad, while streptavidin and goat anti-rabbit antibody were fixed at the test line (T) and control line (C) positions, respectively. **(C)** A positive result was demonstrated by the simultaneous appearance of both the T and C. The appearance of only the C signified a negative. A result was deemed inconclusive or doubtful if only the T appeared. The fluorescence immunoassay analyzer can measure the fluorescence intensity of the T line and the C, and calculate the T/C value.

The recombinase polymerase amplification (RPA)-lateral flow dipstick (LFD) assay is a novel isothermal amplification technique that can achieve amplification of the target gene fragment within 20–40 min at 37–42°C and is often used for the detection of microorganisms and mutated genes (Chang et al., 2024; Hong et al., 2024; Xu et al., 2024). This method eliminates the need for special equipment and complex operational procedures, making it highly suitable for on-site rapid detection. The RPA-LFD assay operates based on isothermal amplification and lateral flow visualization, with detailed principles shown in Figure 1. The 5′ends of the upstream and downstream primers are labeled with fluorescein isothiocyanate (FITC) and biotin, respectively, and the double-labeled product formed after amplification is detected using a dipstick with fluorescent microspheres consisting of highly fluorescent europium (III) nanoparticles (EuNPs). The assay facilitates the visualization of clinical samples, and the long fluorescence decay lifetime of the EuNPs improves the specificity and sensitivity of detection (Du et al., 2022; Xu et al., 2020).

Thus, to address the limitations of existing methods, in this study, based on a DAdV-3 strain (CH-AN-2022) isolated by our research group, DAdV-3-*fiber-2* was used as the target gene to establish a visual fluorescent RPA-LFD rapid detection assay for clinical prevention and control of the virus.

## 2 Materials and methods

### 2.1 Animals

Specific pathogen-free (SPF) duck embryos were purchased from the Chinese Academy of Agricultural Sciences (Harbin, Heilongjiang, China). SPF ducks aged 3 days were incubated and raised at the SPF Animal Incubation Center of Anhui Agricultural University. Ninety 3-day-old Muscovy ducks were procured from a duck farm located in Anhui Province. The ducks were housed in a healthy and controlled setting, with the temperature maintained at 30–34°C in accordance with the age and behavior of the birds.

### 2.2 Establishment of the RPA-LFD assay

#### 2.2.1 Coupling of biomolecules to microspheres

First, the microspheres were washed and activated; 50 μL of highly fluorescent microspheres (100 mg/mL) (Bangs Laboratories, United States) were fully washed twice with 500 μL 4-morpholine ethanesulfonic acid (MES) buffer (10 mM, pH = 6.0); the supernatant was discarded after centrifugation at 4°C at 14,000 × g for 15 min; and the microspheres were resuspended in 200 μL of MES buffer and continuously stirred. Next, 250 μL of 1-ethyl-3-(3-dimethylaminopropyl) carbodiimide hydrochloride (EDC; 10 mg/mL) and N-hydroxysulfosuccinimide sodium salt (NHS) solution (10 mg/mL) were added successively, followed by ultrasound treatment in a water bath for 5 min and dispersed in a thermostatic oscillator at 30°C and 200 rpm for 30 min. Next, the microspheres were coupled to antibodies. The solution was washed twice with 500 μL of phosphate buffer (10 mM, pH 7.4) solution, and the microspheres were resuspended in 1 mL of phosphate buffer. Subsequently, 50 μL of rabbit anti-FITC antibody (Bioss, Beijing, China) was added for coupling. Next, dispersion and washing with phosphate buffer were performed using the methods described above. After washing, the microspheres were resuspended in 500 μL of sealing solution [30 mM ethanolamine and 1% w/v bovine serum albumin (BSA)], dispersed by the same method, and washed in a final wash buffer (sealing solution and 0.05% w/v Tween 20, pH = 8.0) before resuspension in 1 mL of phosphate buffer with 0.1% BSA. Finally, the labeled microspheres were sent to Biohan Biotechnology (Hefei, China) for the preparation and assembly of test dipsticks. Fluorescent microspheres were uniformly sprayed on the bonding pad at 5 μL/cm. The test line (T) was coated with rabbit anti-FITC antibody (Bioss, Beijing, China), and the control line (C) was coated with goat anti-rabbit immunoglobulin G (Artron, Shandong, China) at 1 mg/mL. The dipsticks were stored in a desiccant aluminum foil bag away from light at room temperature.

### 2.3 Design, synthesis, and screening of primers

The full-length sequence of the *fiber-2* gene of DAdV-3 was downloaded from the GenBank database (accession number: OP432083). We designed nine primer pairs (F1/R1, F1/R2, F1/R3, F2/R1, F2/R2, F2/R3, F3/R1, F3/R2, and F3/R3) for the *fiber-2* gene using Oligo 6 software and validated and optimized them using PCR, qPCR, and RPA-LFD, respectively. The primers were synthesized by Sangon Biotech (Shanghai, China), and their sequences are listed in Table 1. First, the PCR reaction system (total volume 20 μL) consisted of the following components: PCR mixture, 10 μL; forward primer (10 μM), 0.5 μL; reverse primer (10 μM), 0.5 μL; DAdV-3 DNA, 1 μL; and double-distilled water (ddH_2_O), 8 μL. The primer pairs were initially validated by PCR to confirm amplification feasibility. Subsequently, the primers were further screened using qPCR. DAdV-3 DNA was diluted 10-, 100-, and 1,000-fold as templates, and qPCR was performed with each sample analyzed in triplicate (mean values were calculated). The primers were deemed qualified if: (i) gel electrophoresis confirmed that the PCR amplification products of the target gene were free of primer dimers; (ii) melting curves analysis showed no non-specific peaks; and (iii) qPCR amplification curves exhibited specific single peaks with the lowest average Ct values. Ultimately, the primer pairs with the highest amplification efficiency were selected for subsequent experiments. This approach was based on the methods employed by other researchers who have performed primer selection for the RPA-LFD assay (Onchan et al., 2022).

**Table 1:** Primers for the RPA method.

Primer	Primer sequences (5′–3′)
DAdV-3-Fiber2-F1	CAATCACTCTCCGTTAGAACTAATCCTCAAG
DAdV-3-Fiber2-F2	CAAGGAGAGAAAGAGTTAGGCATCAACATC
DAdV-3-Fiber2-F3	CACATCGTGCATAACACTAGACAACGGAGGC
DAdV-3-Fiber2-R1	CATACGATCTTGGCATAGTATGCGCACGGAAAC
DAdV-3-Fiber2-R2	CGTAACAGACCCTGCTCCGCAGCACACTTG
DAdV-3-Fiber2-R3	CACACTTGGGCTTGTGTCTTCTGAAGTGTGTC

F, forward primer; R, reverse primer.

The RPA assay was performed according to the manufacturer’s instructions (LeSun Bio, Wuxi, China). The reaction mixtures contained 25 μL of reaction buffer, 16 μL of ddH_2_O, lyophilized enzyme powder, 2 μL of the FITC-labeled forward primer (10 μM), 2 μL of the biotin-labeled reverse primer (10 μM), 2 μL of pMD18-T-*fiber-2* recombinant vector template, and 3 μL of activator. After all the reaction components were added, we performed manual flicking and brief centrifugation. The amplification was performed at 37°C for 20 min. The amplified product was then diluted 10 times with 1% skim milk powder and tested with the dipstick. After 5–10 min, the results were observed (Figure 1). A positive result was demonstrated by the simultaneous appearance of both the T and C lines. Conversely, a negative result was evidenced by the appearance of only the C line, which indicated the absence of the target gene fragment. The results were deemed inconclusive or doubtful if only the T line appeared.

### 2.4 Screening of optimal reaction conditions for RPA-LFD assays

The optimum conditions of the above assay were further optimized, and the optimum buffer type, temperature, and dilution ratio were screened. The 70 μL diluted amplified product was added to the dipsticks. After 5–10 min, the fluorescence signal intensity of the C and T lines was read by a fluorescence immunoassay analyzer (HIT-91A, Biouhan, Hefei, China), and the T/C value was calculated. Each group of experiments was repeated 3 times to calculate the mean (M) ± standard deviation (SD).

#### 2.4.1 Optimization of the buffer type

The amplified product was diluted 10-fold with phosphate-buffered saline with Tween 20 (PBST), 1% skim milk powder, or Blocker^™^ Casein in phosphate-buffered saline (PBS; Thermo Fisher, United States) and then added to the dipsticks.

#### 2.4.2 Optimization of temperature

To identify the optimal reaction temperature, the RPA reaction was performed at six temperatures (30°C, 33°C, 36°C, 39°C, 42°C, and 45°C) controlled by the PCR instrument. At the end of the reactions, the RPA product was diluted 10-fold with 1% skim milk powder.

#### 2.4.3 Optimization of the dilution ratio

Highly viscous amplification products must be appropriately diluted to allow testing of the target gene amplification products. At the end of the reaction, the RPA amplification products were diluted 5-, 10-, 20-, 30-, 40-, 50-, 60-, 70-, and 80-fold in 1% skim milk powder (Table 2).

**Table 2:** Dilution ratio of RPA products.

Reagent name	Dilution ratio								
	5	10	20	30	40	50	60	70	80
RPA products (μL)	14.00	7.00	3.50	2.33	1.75	1.40	1.17	1.00	0.88
1% skim milk powder (μL)	56.00	63.00	66.50	67.67	68.25	68.60	68.83	69.00	69.12

### 2.5 Analysis of the specificity, sensitivity, and limit of blank of the RPA-LFD assay

The specificity, sensitivity, and limit of blank (LOB) of the RPA-LFD assay were also verified. The detection method for the amplified products was the same as described above in section 2.3.

#### 2.5.1 Specificity analysis

The DNA of DAdV-3, FAdV-4, DuCV-1 (Duck circovirus-1), DAdV-2, FAdV-8a, and the cDNA of MDPV (Muscovy duck parvovirus), DHV (Duck hepatitis virus), H7, and H9 subtypes of AIV (Avian Influenza virus) were used as templates for RPA specificity analysis to identify cross-reactions with other viruses in the assay. At the end of the reaction, the RPA amplification products were diluted 10-fold with 1% skim milk powder. The experiment was repeated 3 times to calculate the M ± SD.

#### 2.5.2 Sensitivity analysis

Serial dilutions of pMD18-T-*fiber-2* plasmid standards ranging from 10^6^ to 10^0^ copies/μL were used as templates. Optimized conditions for the RPA-LFD assay were used in the sensitivity test. The experiment was repeated 3 times to calculate the M ± SD.

#### 2.5.3 LOB analysis

The RPA assay system was tested with 20 samples of 1% skim milk powder buffer; the results were recorded on a fluorescence immunoassay analyzer; and the data were collated and analyzed. The formula used to calculate LOB was as follows: M + 2 × SD.

### 2.6 Clinical sample analysis

To evaluate the concordance rate between the RPA-LFD assay and qPCR, 65 cloacal samples collected from three duck farms with clinical outbreaks were tested using both the established RPA-LFD assay and qPCR, and their results were compared. The qPCR assay was performed following a previously reported protocol (Zhang X. et al., 2024). The cycle threshold (Ct = 37), derived from amplification of the positive control plasmid pMD18-T-DAdV-3-*fiber-2* at a concentration of 1 × 10^2^ copies/μL, was defined as the cutoff value. Results were interpreted as follows: samples were classified as positive if the measured Ct value was less than 37, and negative if the measured Ct value exceeded 37. Each sample was analyzed in triplicate, and the mean Ct value was computed for final interpretation. The consistency between the two methods was evaluated by calculating the Kappa (κ) coefficient and *p*-value. κ is used to measure the degree of consistency in the data, and its value ranges between −1 and 1; additionally, the *p*-value represents the probability that the observed consistency occurred by chance. The closer the κ value is to 1, the higher the consistency between the indicators; on the other hand, the smaller the *p*-value, the more significant the consistency between the indicators. The formula for calculating the relevant detection indicators is presented below, in which a, b, c, and d are the numbers of samples counted, and n is the total number of samples: κ = (P_o_ − P_e_)/(1 − P_e_), P_o_ = (a + d)/(a + b + c + d), P_e_ = [(a + c)(b + d) + (a + b)(c + d)]/(a + b + c + d)^2^, where P_o_ is the actual coincidence rate, P_e_ is the theoretical coincidence rate, and *n* is the total number of samples (Table 3).

**Table 3:** Chi-square test was conducted on a four-grid table of data.

Method	Sample	qPCR		
		Positive	Negative	Total
RPA-LFD	Positive	a	b	a + b
	Negative	c	d	c + d
	Total	a + c	b + d	a + b + c + d

(a) Both qPCR and RPA-LFD positive. (b) qPCR negative and RPA-LFD positive. (c) qPCR positive and RPA-LFD negative. (d) Both qPCR and RPA-LFD negative.

### 2.7 Clinical detection

To validate the efficacy of the timely detection with RPA-LFD for the effective prevention and control of DAdV-3 infections in a clinical setting, this study was also conducted in a farm. Thirty 3-day-old SPF ducks were inoculated with 0.2 mL of duck embryo allantoic fluid containing 2 × 10^5^ TCID_50_/0.1 mL by subcutaneous injection on the neck and back. Two days later, the infected ducklings were marked and randomly assigned to two groups and placed among 45 3-day-old healthy Muscovy ducks, designated as Group 1 and Group 2. After 4 days of mixed culture, the SPF ducks were culled based on the markers and euthanized. During the mixed culture period, if the ducklings in Group 1 showed poor mental conditions or mild diarrhea symptoms, cloacal swabs were collected and promptly tested using RPA-LFD, and positive Muscovy ducks were culled. Group 2 was fed in accordance with normal procedures without any detection and culling, and the records of the ducklings were maintained for 30 days. The determination of virus TCID_50_ and the handling of the experimental animals were performed as previously reported (Zhang X. et al., 2024).

## 3 Results

### 3.1 Screening of the best primer pair for RPA

**Figure 2:**
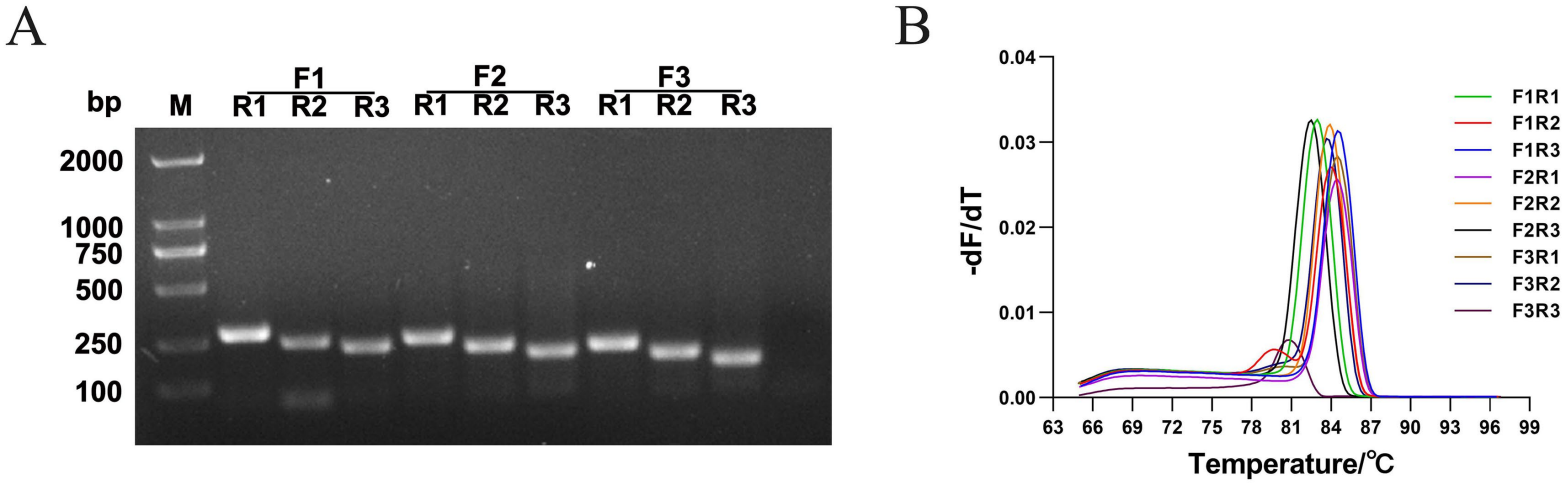
Screening of optimal primer pairs. **(A)** Nine primer pairs (F1R1, F1R2, F1R3, F2R1, F2R2, F2R3, F3R1, F3R2, and F3R3) for PCR amplification, and the amplified fragments were visualized by 1.5% agarose gel electrophoresis. The target fragment sizes were 275, 242, 220, 244, 215, 190, 212, 180, and 158 bp, respectively. **(B)** qPCR reactions with nine primer pairs, obtaining the melt curves (DAdV-3 DNA 1:1,000 dilution). The data are presented as the mean values (*n* = 3).

PCR, qPCR, and RPA-LFD were used to select the optimal primer pairs. First, we performed PCR verification using the nine pairs of primers designed in this study. After electrophoresis on a 1.5% agarose gel, we observed the clearest bands when using 1 μL of the primers, as shown in Figure 2A. All nine primer pairs (F1/R1: 275 bp; F1/R2: 242 bp; F1/R3: 220 bp; F2/R1: 244 bp; F2/R2: 215 bp; F2/R3: 190 bp; F3/R1: 212 bp; F3/R2: 180 bp; F3/R3: 158 bp) could amplify the target fragment and yielded products with the expected size; however, the F1/R2, F1/R3, F2/R2, F2/R3, F3/R2, and F3/R3 amplification products had obvious primer dimers. In contrast, F1/R1, F2/R1, and F3/R1 produced clear bands without primer dimers.

Furthermore, 10-, 100-, and 1,000-fold DNA dilutions were used as templates, and qPCR was used to evaluate the primers. The amplification efficiencies of the different primers are shown in Table 4. When F1/R1 was used as the primer pair, the amplification efficiency was the highest. Analysis of the melting curves revealed that F1/R2 and F3/R3 showed non-specific amplification (Figure 2B). Nine primer pairs were used to amplify the gene product using RPA, and the results showed that the amplified products of F1/R1, F2/R1, F3/R3, and F3/R1 showed the clearest bands on the dipsticks.

**Table 4:** qPCR Ct values of DAdV-3 DNA at different dilutions.

Primer	qPCR		
	10-fold	100-fold	1,000-fold
F1R1	18.28	23.73	27.58
F1R2	18.71	23.48	26.92
F1R3	19.50	23.22	27.17
F2R1	19.36	23.74	27.83
F2R2	18.71	23.38	27.21
F2R3	18.34	23.24	27.32
F3R1	19.35	23.69	27.75
F3R2	19.08	23.50	27.68
F3R3	17.96	23.34	27.76

In summary, the primer pair F1/R1 was chosen as the optimal pair for the RPA-LFD assay.

### 3.2 Optimization of RPA condition

**Figure 3:**
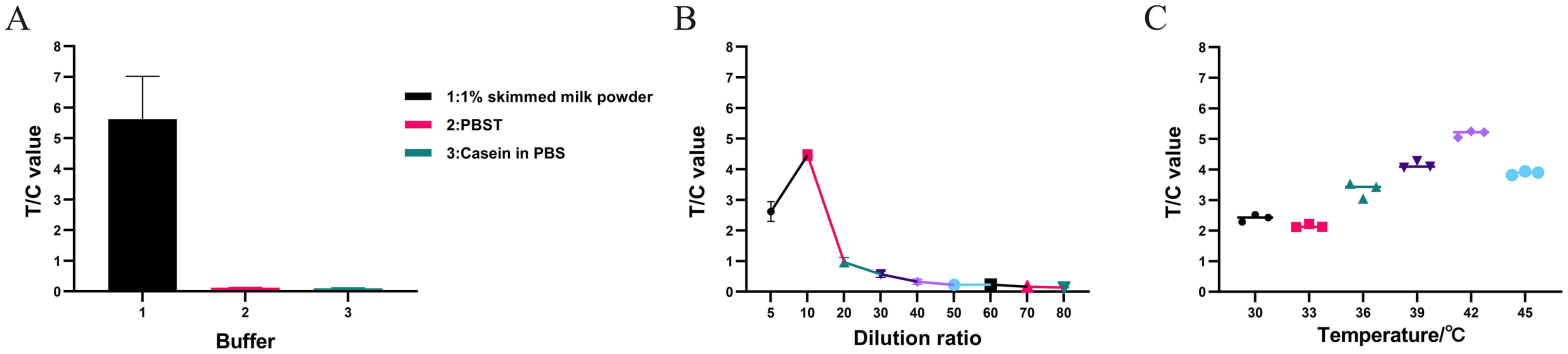
Screening of optimal buffer types, dilution ratio, and temperature in RPA assay. **(A)** The RPA amplification product was diluted using 1% skimmed milk powder, PBST, and Blocker^™^ Casein in PBS. The fluorescence immunoassay analyzer can measure the fluorescence intensity of the T and C, and calculate the T/C value. **(B)** Dilute the RPA amplification product with 1% skim milk powder to 5-, 10-, 20-, 30-, 40-, 50-, 60-, 70-, and 80-fold, respectively, and the resulting T/C values are 2.6214, 4.4628, 0.9683, 0.5707, 0.3302, 0.2247, 0.2366, 0.1646, and 0.1415, respectively. **(C)** The T/C values were measured at different RPA amplification temperatures (30, 33, 36, 39, 42, and 45°C). The fluorescence immunoassay analyzer can measure the fluorescence intensity of the T and C, and calculate the T/C value. All data in **A–C** are presented as M ± SDs (*n* = 3).

The optimal conditions for highly efficient RPA were determined by evaluating different buffer types, temperatures, and dilution ratios. The amplified products were diluted 10-fold with 1% skim milk powder, PBST, and Blocker^™^ casein in PBS. As shown in Figure 3A, the mean T/C values with the three diluents were 5.6183, 0.115667, and 0.0995, respectively. Therefore, 1% skim milk powder was identified as the optimal diluent for the RPA products.

RPA products were diluted at gradients of 5- to 80-fold as shown in Table 2. The results are shown in Figure 3B. When the products were diluted 5-, 10-, 20-, 30-, 40-, 50-, 60-, 70-, and 80-fold, the mean T/C values were 2.6214, 4.4628, 0.9683, 0.5707, 0.3302, 0.2247, 0.2366, 0.1646, and 0.1415, respectively. The RPA products had relatively small T/C values at dilutions greater than 20-fold. A 10-fold dilution resulted in the highest T/C value. Therefore, 10-fold dilution was identified as the optimal dilution level for RPA products.

The RPA reaction was also performed within a specific temperature range. The results are shown in Figure 3C. The amplification efficiency tended to increase from 33 to 42°C, while it decreased from 42 to 45°C. Thus, the optimal amplification temperature for the RPA-LFD assay was identified as 42°C.

### 3.3 Sensitivity, specificity, and LOB determination of the RPA-LFD assay

**Figure 4:**
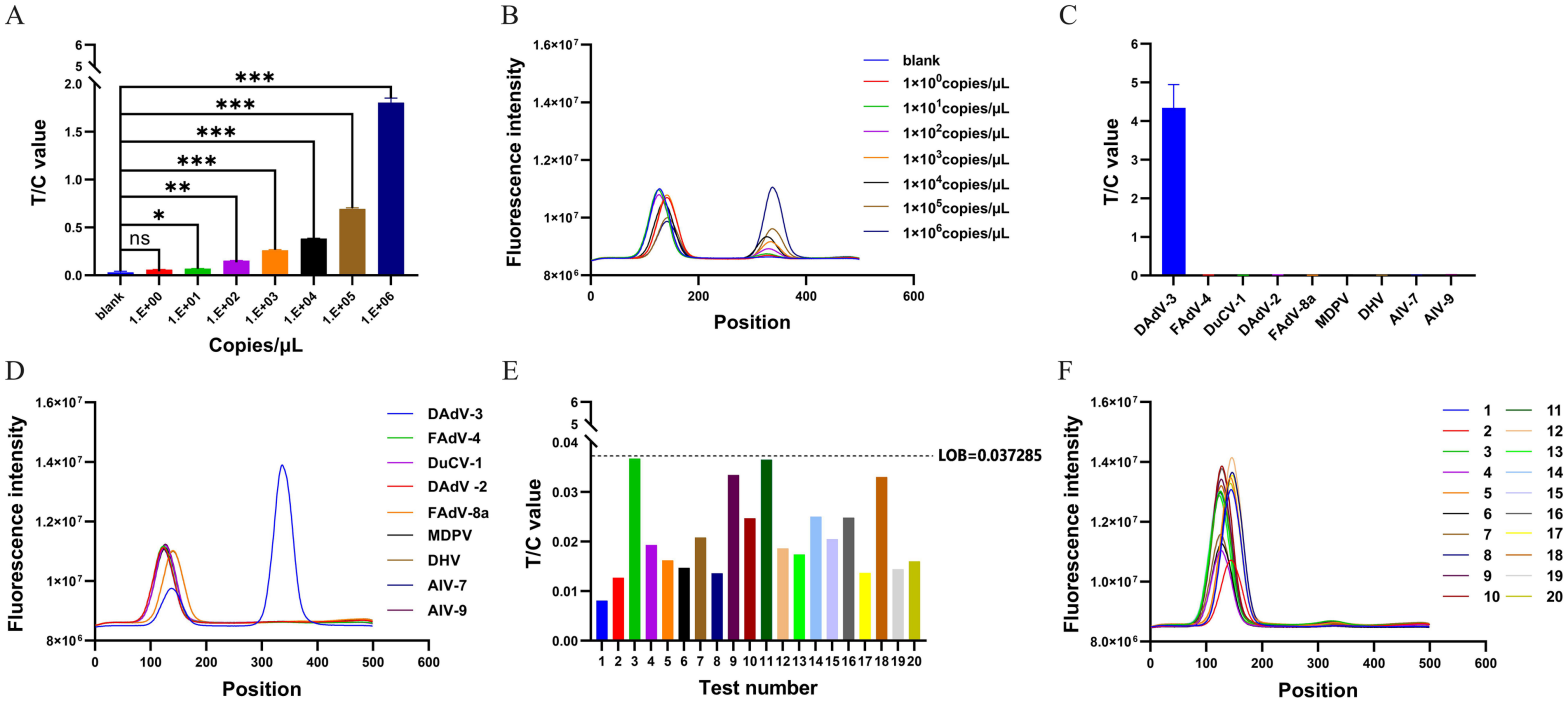
Performance analysis of the RPA-LFD assay. **(A)** The RPA-LFD assay is employed to detect the values of T/C under different concentrations of pMD18-T-*fiber-2* plasmid (10^0^–10^6^ copies/μL) The data are presented as M ± SDs (*n* = 3). ^***^p < 0.0001, ^**^p < 0.001, ^*^p < 0.01, and ^ns^*p* > 0.05. **(B)** Representative dipsticks for plasmid concentrations in **A**. The fluorescence signal intensity of T and C lines are calculated by a fluorescence immunoassay analyzer. The position of the C value is between 60 and 160, and the position of the T value is between 320 and 420. **(C)** The specificity of the RPA-LFD assay was assessed using FAdV-4, DuCV-1, DAdV-2, FAdV-8a, MDPV, DHV, AIV-H7, and AIV-H9 viral nucleic acids. The T/C value is calculated by a fluorescence immunoassay analyzer. The data are presented as M ± SDs (*n* = 3). **(D)** Specificity strip readouts: corresponding to the LFD results for the viruses in **C**. The position of the C value is between 60 and 160, and the position of the T value is between 320 and 420. **(E)** To determine the LOB for the RPA-LFD assay, a set of 20 1% skim milk powder buffer samples were employed as amplification templates for assay, and read the T/C values. Based on the calculation LOB = M + 2SD, the result obtained is 0.037285. **(F)** Detect the fluorescence signal intensity of T and C for the amplification products of 20 1% skim milk powder buffer samples. The position of the C value is between 60 and 160, and the position of the T value is between 320 and 420.

The sensitivity of the RPA-LFD assays was evaluated using the pMD18-T-*fiber-2* plasmid diluted to concentrations ranging from 10^6^ to 10^0^ copies/μL. The 1 × 10^1^ copies/μL sample showed a significantly higher T/C value compared to the negative control (Figures 4A,B). The detection limit for the RPA-LFD assay was 1 × 10^1^ copies/μL. The specificity of the RPA-LFD assay was tested using nucleic acids of FAdV-4, DuCV-1, DAdV-2, FAdV-8a, MDPV, DHV, AIV-H7, and AIV-H9. Only DAdV-3 yielded positive signals (both T and C lines) in the RPA-LFD assay. Notably, FAdV-4, DuCV-1, DAdV-2, FAdV-8a, MDPV, DHV, AIV-H7, and AIV-H9 did not show cross-reactivity (Figures 4C,D), indicating that the established RPA-LFD assay had excellent specificity. LOB analysis was performed to determine the lowest signal intensity distinguishable from blank samples. Using 20 blank samples, the LOB of the RPA-LFD assay was calculated as 0.037285 based on the formula LOB = M + 2SD (Figures 4E,F).

### 3.4 Validation of the RPA-LFD assay by using clinical samples

**Figure 5:**
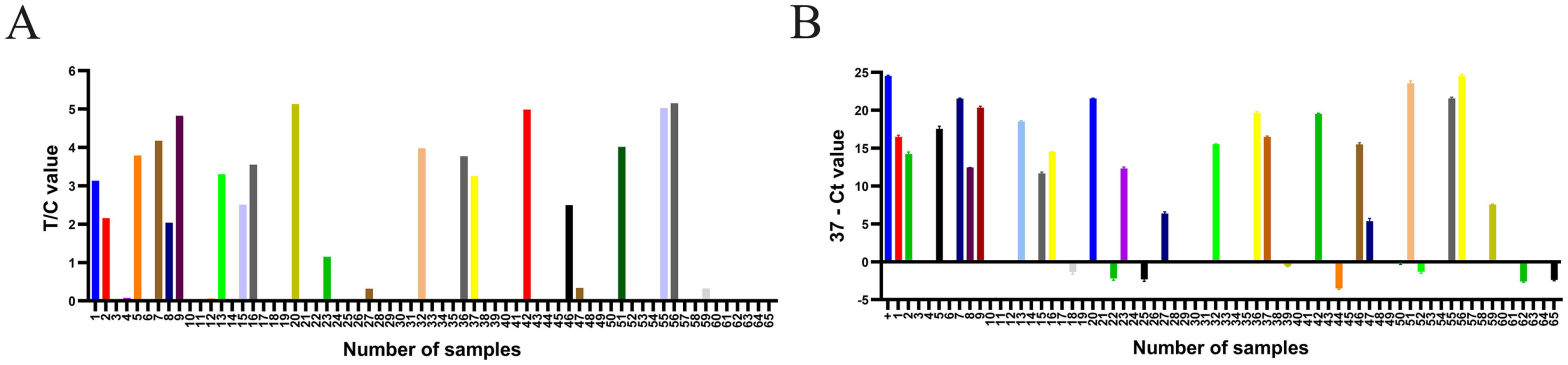
The RPA-LFD and qPCR assays were employed to analyze a total of 65 clinical samples. **(A)** The T/C values were obtained by detecting 65 samples using the RPA-LFD assay. **(B)** Ct values were obtained through qPCR analysis of 65 samples. The “+” sample is a positive plasmid. A sample was considered positive when the difference between 37 and the cycle threshold (Ct value; 37-Ct) was greater than 0, and negative when the difference was less than 0. The data are presented as the means ±SDs (*n* = 3).

The established RPA-LFD was used to evaluate 65 suspected DAdV-3 cloacal swab samples (Figure 5A), and the results for all samples were verified using qPCR (Figure 5B). As shown in Table 5, the positivity rate of the RPA-LFD assay was 33.8% (22/65), while the positivity rate in qPCR assessments was also 33.8% (22/65), with both methods showing a coincidence rate of 100% (65/65). The κ value of the RPA-LFD and qPCR assays was 1 (*p* < 0.001), indicating almost perfect agreement between the two methods.

**Table 5:** Detection results of 65 clinical samples by RPA-LFD and qPCR assays.

	Sample	qPCR			Kappa (κ)	*p*-value of kappa
		Positive	Negative	Total		
RPA-LFD	Positive	22	0	22	1	<0.001
	Negative	0	43	43		
	Total	22	43	65		

### 3.5 Detection and analysis of the clinical efficacy of the RPA-LFD assay

In Group 1, four DAdV-3-positive Muscovy ducks identified by RPA-LFD were culled within the first 10 days of the 30-day co-infection period [mortality rate, 8% (4/45)]. In contrast, in Group 2, after 30 days of observation, nine ducks died [mortality rate, 20% (9/45)], and the final positive Muscovy duck was identified on day 28. These results indicate that timely detection and culling substantially reduced the mortality rate, from 20 to 8%.

## 4 Discussion

The clinical experimental findings of this study highlighted the critical role of timely intervention in controlling DAdV-3 infections. Specifically, the mortality rate of DAdV-3-infected Muscovy ducks reached 20%, while their long-term sporadic death rate was significantly lower than that of SPF ducklings, likely due to the higher susceptibility of SPF ducklings to the virus. Importantly, prompt isolation of sick ducks reduced the mortality rate to 8%, underscoring that timely detection and isolation are pivotal for preventing and controlling DAdV-3. Against this backdrop, the development of a rapid and reliable detection method is imperative. Existing assays for the detection of DAdV-3 cannot simultaneously meet the requirements of high specificity and ease of operation (Ather et al., 2024; Wan et al., 2018; Xie et al., 2011). In contrast, the sensitivity of our newly developed RPA-LFD assay was as high as 1 × 10^1^ copies/μL, and the detection results could be obtained within 30 min. This level of sensitivity is comparable to that of qPCR (Table 5), indicating the potential of the RPA-LFD assay as a new approach for clinical DAdV-3 detection. This method has also been applied by researchers to test the sensitivity and specificity for other avian viruses; for instance, the minimum detection limits for infectious bursal disease virus (IBDV), AIV (H5, H7, and H9 subtypes), and Newcastle disease virus (NDV) were 10^1^, 10^2^, and 10^2^ copies/μL, respectively, with no cross-reactivity observed (Wang et al., 2023; Wang et al., 2020; Zhang Z. et al., 2024). In the current study, 65 cloacal samples tested in this study were collected from three duck farms experiencing clinical outbreaks as suspected positive specimens, which may explain the relatively high positivity rate (33.8%).

Beyond method validation, our analysis of detection targets and viral tropism provided additional insights. The *fiber-2* gene was selected as the target due to its high conservation across all DAdV-3 strains, ensuring the assay’s applicability to diverse DAdV-3 isolates. Consistent with reports that the intestine is the primary excretory organ of fowl adenoviruses (including DAdV-3) (Shi et al., 2022; Zhang X. et al., 2024), cloacal swabs effectively indicated viral presence in our study. Notably, positive results in this study suggest that DAdV-3 is not only detected in Muscovy ducks but also in different breeds of elderly ducks, including 180-day-old Peking ducks, 240-day-old white ducks, and 200-day-old Shaoxing ducks, indicating that DAdV-3 can spread not only within Muscovy ducks but also across breeds. These results suggest that DAdV-3 can infect not only ducklings but also older ducks; however, the specific transmission mechanism requires further study.

This study has certain limitations. First, the efficacy of the assay was validated under controlled experimental conditions, which may fail to fully recapitulate the intricate nature of natural clinical settings-encompassing variations in pathogen load, fluctuations in host immune competence, and co-infections prevalent in poultry production systems. Second, the number and diversity of duck breeds employed for validation were relatively restricted, potentially compromising the statistical power and generalizability of the findings. Furthermore, the current cost of the method remains relatively high, which may impede its widespread adoption in resource-limited field settings. Consequently, the clinical applicability and robustness of the assay including its performance under field conditions and consistent detection across diverse duck populations-warrant further validation, while cost optimization is also imperative. Notably, the optimized RPA-LFD system holds promise for extension to the detection and clinical elimination of other avian pathogens (including viruses and bacteria), with particular potential utility in the screening and culling of oncogenic pathogens.

## Data Availability

The original contributions presented in the study are included in the article/supplementary material, further inquiries can be directed to the corresponding author.
